# Efficacy and safety of dupilumab following discontinuation of JAK inhibitors in patients with moderate-to-severe atopic dermatitis: a single-center retrospective study

**DOI:** 10.3389/fimmu.2026.1841762

**Published:** 2026-07-01

**Authors:** Qian Wang, Ge Yang, Ying Li, Jing Xue, Lixia Zhang

**Affiliations:** Institute of Dermatology and Venereology, Sichuan Provincial People’s Hospital, School of Medicine, University of Electronic Science and Technology of China, Chengdu, China

**Keywords:** atopic dermatitis, dupilumab, efficacy, Janus kinase inhibitor, safety

## Abstract

**Background:**

Switching systemic therapies is common in atopic dermatitis (AD). Current reports focus mainly on switching from dupilumab to janus kinase inhibitors (JAKi), with limited data on transitioning from JAKi to dupilumab.

**Objective:**

To explore the feasibility of dupilumab as maintenance or replacement therapy following JAKi discontinuation in moderate-to-severe AD.

**Methods:**

This single-center retrospective study enrolled patients with moderate-to-severe AD between June 2023 and January 2026 who initially received JAKi and replaced with dupilumab. Clinical scores (EASI, PP-NRS, DLQI, and ADCT) were analyzed at baseline, JAKi discontinuation, dupilumab initiation, and week 16. Safety events were recorded.

**Results:**

Fifteen patients aged 17–81 years (41.93 ± 20.90) were included. At the time of JAKi discontinuation, 10 patients (66.67%) achieved EASI-75. Nine patients switched immediately to dupilumab, and 6 switched after 2–20 weeks. After 16 weeks of dupilumab treatment, 14 patients (93.33%) demonstrated clinical benefit (achieved EASI-50, PP-NRS ≤ 4, DLQI ≤ 5, and ADCT < 7), of whom 11 (73.33%) achieved EASI-75. Among the 9 immediate-switch patients, 4 experienced recurrence within the first 4 weeks; 3 stabilized with short-term conventional therapy, and 1 resumed upadacitinib after 8 weeks. Adverse events were reported more frequently with JAKi (7/15, 46.67%) than with dupilumab (0/15, 0.00%); no serious adverse events were observed.

**Conclusion:**

Dupilumab may be a potential maintenance or alternative treatment option for moderate-to-severe AD patients who require discontinuation of JAKi therapy. Disease fluctuations after direct switching warrant attention, and larger prospective studies are needed to validate switching strategies.

## 1 Introduction

Atopic dermatitis (AD) is a chronic, recurrent inflammatory skin disease characterized by intense pruritus, eczematous lesions, skin barrier dysfunction, and abnormal immune responses. It severely impairs patients’ quality of life and imposes a significant economic burden. For patients with moderate-to-severe AD, topical treatments alone have limited efficacy, making systemic therapy necessary. In the past, glucocorticosteroids and immunosuppressants such as cyclosporine were the primary systemic therapeutic options. However, these treatments have insufficient efficacy and notable adverse effects, making them unsuitable for long-term management. In recent years, advances in understanding the disease pathogenesis and breakthroughs in immunopharmacology have ushered AD treatment into a new era of targeted therapy. While increased treatment options benefit both physicians and patients, they may also complicate the selection of the optimal therapeutic approach.

Currently, dupilumab (a biologic) and the selective Janus kinase inhibitors (JAKi) upadacitinib and abrocitinib are approved systemic therapies for moderate-to-severe atopic dermatitis by the National Medical Products Administration (NMPA) in China, and have become widely used in clinical practice. A series of clinical studies have demonstrated their clinical efficacy in moderate-to-severe AD, though each has distinct advantages in disease control and safety. JAKi offer faster symptom relief but carry higher safety risks including infections, thromboembolism and laboratory abnormalities (1), while dupilumab has a favorable safety profile especially suitable for patients with severe comorbidities, infants, and the elderly (2, 3). When formulating treatment plans, physicians must thoroughly consider individual patient characteristics and drug-specific differences, engaging in shared decision-making with patients. Clinical practice has observed in patients who experience adverse reactions (such as ocular and/or facial adverse events) or inadequate response to dupilumab, switching to upadacitinib or abrocitinib may resolve these adverse events and potentially yield superior therapeutic outcomes (4). However, for chronic diseases like AD, in addition to rapid symptom control, long-term maintenance therapy is essential, making safety particularly critical. Potential safety concerns associated with JAKi, particularly at high doses, continue to raise concerns among both physicians and patients regarding their long-term use. Therefore, it is necessary to explore whether dupilumab can serve as a maintenance or alternative therapy after discontinuation of JAK inhibitors. Currently, relevant research in this area is limited. This study retrospectively analyzed the efficacy and safety of 15 patients with moderate-to-severe AD who switched from JAKi to dupilumab, aiming to preliminarily explore the feasibility of dupilumab as maintenance or alternative therapy after JAKi withdrawal, thereby providing preliminary real-world evidence for this switching strategy.

## 2 Methods

### 2.1 Subjects

This retrospective study analyzed patients with moderate-to-severe AD treated at Sichuan provincial people’s hospital from June 2023 to January 2026. AD diagnosis was based on the Hanifin and Rajka criteria (5). Inclusion criteria were: 1) moderate-to-severe disease severity (SCORAD ≥ 25); 2) age ≥12 years; 3) initial treatment with JAKi, followed by switching to standard-dose dupilumab therapy (600 mg/400 mg initial dose, then 300 mg/200 mg every 2 weeks) with a 16-week observation period. The absence of either the clinician−reported outcome (EASI) or all patient−reported outcomes (PP−NRS, DLQI, and ADCT) served as an exclusion criterion.AD relapse was defined according to the the European Task Force on Atopic Dermatitis (ETFAD) criteria: “an acute, clinically significant worsening of the signs and symptoms of AD that requires therapeutic intervention” (6). This study was approved by the Ethics Committee of Sichuan Provincial People’s Hospital (No. 2022-327). Since this study is retrospective, all data were anonymized prior to analysis, and no personally identifiable information was involved. Moreover, no intervention or additional contact with patients was conducted during the study. Therefore, the ethics committee approved a waiver of informed consent. The study was performed in compliance with the principles of the Declaration of Helsinki.

### 2.2 Data collection

Patient data were collected from medical records including age, sex, AD duration, atopic comorbidities, family history of atopy, prior medications, underlying diseases, AD-related clinical scores [eczema area and severity index (EASI), peak pruritus numerical rating scale (PP-NRS), dermatology life quality index (DLQI), and atopic dermatitis control tool (ADCT) at baseline, JAKi discontinuation, dupilumab initiation, and week 4, 8, 16 on dupilumab], adverse events, reasons for JAKi discontinuing, the time interval between JAKi discontinuation and dupilumab initiation, etc. All improvements in clinical scores were calculated relative to baseline. Data collection was performed independently by two experienced dermatologists (QW and GY). Any disagreements were resolved through discussion or, when necessary, by consulting a third senior dermatologist (LZ).

### 2.3 Statistical analyses

All data were analyzed using SPSS version 26.0 (IBM, New York, NY, USA). Descriptive analysis was employed to examine demographic data and clinical characteristics. Continuous variables were expressed as mean ± standard deviation, and categorical variables were described as percentages. The Shapiro-Wilk test was used to assess whether the differences between paired measurements followed a normal distribution. If the differences were normally distributed, a paired t-test was applied; otherwise, the Wilcoxon signed-rank test was used. p <0.05 was considered statistically significant.

## 3 Results

### 3.1 Patient characteristics at baseline

Based on the inclusion and exclusion criteria, this study included a total of 15 patients and excluded 9 patients. Of the 15 patients, 11 were male and 4 were female, with ages ranging from 17 to 81 years (41.93 ± 20.90). The disease duration of AD was 0.5–11 years (4.77 ± 4.21). Ten patients (66.67%) presented with atopic comorbidities, including 7 cases of allergic rhinitis, 1 case of asthma, 1 case of allergic rhinitis with concomitant asthma, and 1 case of allergic rhinitis and allergic conjunctivitis. Three patients (20.00%) had a family history of atopy. All patients had previously used antihistamines and topical corticosteroid; 4 had used *Tripterygium wilfordii* Hook F, and 2 had used systemic corticosteroids. None had received systemic immunosuppressants or phototherapy. Baseline scores for EASI, PP-NRS, DLQI, and ADCT were (17.40 ± 7.59), (7.27 ± 1.44), (15.67 ± 5.54), and (18.40 ± 4.01), respectively. The Clinical characteristics and demographic data are summarized in **Table 1**.

**Table 1:** Clinical characteristics and demographical data at baseline.

Characteristics	Value [n (%)]
Sex, male	11 (73.33)
Age, year	41.93 ± 20.90
AD durations, years	4.77 ± 4.21
Clinical scores	
EASI	17.40 ± 7.59
PP-NRS	7.27 ± 1.44
DLQI	15.67 ± 5.54
ADCT	18.40 ± 4.01
Atopic comorbidities	
Allergic rhinitis	7 (46.67)
Asthma	1 (6.67)
Allergic rhinitis+Asthma	1 (6.67)
Allergic rhinitis+Allergic conjunctivitis	1 (6.67)
Family history of atopy	3 (20.00)
Other comorbidities	
Hypertension	1 (6.67)
CSU	1 (6.67)
Hypothyroidism	1 (6.67)
CKD stage 3	1 (6.67)
Previous systemic treatment	
Systemic antihistamines	15 (100.00)
*Tripterygium wilfordii* Hook F	4 (26.67)
Systemic glucocorticoids	2 (13.33)

AD, atopic dermatitis; EASI, eczema area and severity index; PP-NRS, peak pruritus numerical rating scale; DLQI, dermatology life quality index; ADCT, atopic dermatitis control tool, CSU, chronic spontaneous urticaria; CKD, chronic kidney disease.

### 3.2 JAKi treatment status

Nine patients received upadacitinib 15 mg qd and 6 patients received abrocitinib 100 mg qd as initial treatment doses; none escalated to higher doses. JAKi treatment duration ranged from 4 to 66 weeks (32.67 ± 18.79). At the time of JAKi withdrawal, all clinical scores showed significant reduction compared to baseline [EASI (17.40 ± 7.59 vs. 5.41 ± 4.47, p=0.000), PP-NRS (7.27 ± 1.44 vs. 2.47 ± 1.60, p=0.000), DLQI (15.67 ± 5.54 vs. 4.40 ± 3.81, p=0.000), ADCT (18.40 ± 4.01 vs. 5.47 ± 3.46, p=0.000)]. Among the patients, 13 (86.67%) achieved EASI-50, 10 (66.67%) achieved EASI-75, 13 (86.67%) achieved PP-NRS ≤4, and 11 (73.33%) achieved DLQI ≤5 and ADCT <7; 2 (13.33%) patients did not achieve EASI-50. The primary reasons for switching to dupilumab included adverse reactions, insufficient efficacy, and concerns about the safety of JAKi among physicians or patients. Nine patients switched to dupilumab immediately after JAKi discontinuing, while 6 patients switched after an interval of 2–20 weeks after discontinuation.

### 3.3 Disease status after switching to dupilumab

Of the 9 patients who were immediately switched to dupilumab, 8 had achieved EASI-50 at the time of drug switch (including 6 who had achieved EASI-75), while 1 had not achieved EASI-50. Four patients with EASI-75 response transitioned smoothly without significant signs of relapse during the switch and maintained EASI-75, PP-NRS ≤4, DLQI ≤5, and ADCT <7 at 16 weeks of dupilumab treatment. Another 4 patients experienced recurrence within the first 4 weeks after switching. Among them, 3 patients (1 with EASI-75 response, 2 with EASI-50 response) gradually stabilized after receiving adjunctive therapy with antihistamines, *Tripterygium wilfordii* Hook F, and topical corticosteroids for 2–4 weeks, and regained EASI-50/EASI-75, PP-NRS ≤4, DLQI ≤5, and ADCT <7 after continuing dupilumab until 16 weeks. The remaining patient, who originally had an EASI-75 response, remained uncontrolled despite the addition of the above conventional therapies and resumed upadacitinib treatment after 8 weeks. One patient who had not achieved EASI-50 at the time of JAKi discontinuation achieved EASI-75 after switching to dupilumab for 16 weeks.

Among the 6 patients who switched to dupilumab after discontinuing JAKi for 2–20 weeks, 5 had achieved EASI-50 at the time of JAKi withdrawal (4 had reached EASI-75), while 1 had not reached EASI-50. The 5 patients with EASI-50 response all relapsed within 2–20 weeks after discontinuation and required re-initiation of systemic therapy. At the time of switching to dupilumab, the EASI, PP-NRS, DLQI, and ADCT scores of the 6 patients were (15.47 ± 5.01), (6.00 ± 1.79), (8.00 ± 2.10), and (13.83 ± 4.17), respectively. After 16 weeks of dupilumab treatment, all 6 patients achieved EASI-50, PP-NRS ≤4, DLQI ≤5, and ADCT <7, with 5 achieving EASI-75.

In summary, after discontinuing JAKi and following 16 weeks of dupilumab treatment, 14 out of 15 patients (93.33%) achieved EASI-50, PP-NRS ≤4, DLQI ≤5, and ADCT <7, among whom 11 (73.33%) achieved EASI-75.

### 3.4 Safety

During JAKi treatment, 7 patients (46.67%) experienced adverse events: 4 patients (26.67%) demonstrated positive γ-interferon release tests without evidence of active tuberculosis, 1 patient (6.67%) had elevated liver enzymes, 1 patient (6.67%) had elevated creatinine, and 1 (6.67%) patient experienced worsening acne. All events were mild to moderate in severity. No safety events were reported after switching to dupilumab.

**Figure 1:**
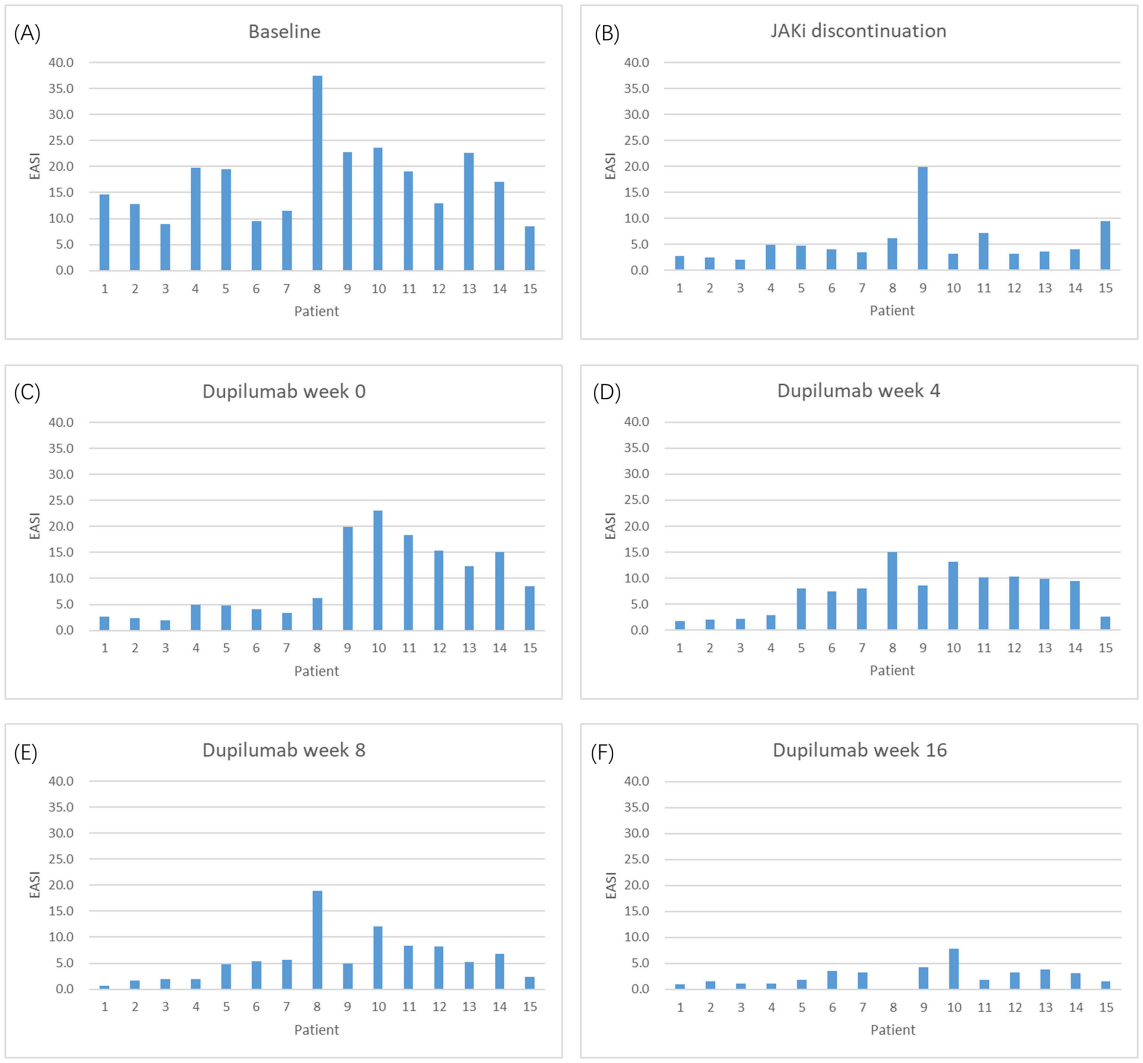
EASI scores of each patient at baseline **(A)**, JAKi discontinuation **(B)**, dupilumab initiation **(C)**, and 4 **(D)**, 8 **(E)**, and 16 weeks **(F)** of dupilumab treatment. Patients 1–9 underwent immediate switching: patients 1–4 transitioned smoothly and maintained EASI-75; patients 5–8 experienced relapse within the first 4 weeks after switching, with patient 8 resuming upadacitinib after 8 weeks; patient 9 had an inadequate response to JAKi and achieved EASI-75 after 16 weeks on dupilumab. Patients 10–15 switched 2–20 weeks after discontinuing JAKi: patients 10–14 had achieved EASI-75 or EASI-50 at the time of JAKi discontinuation but all relapsed within 2–20 weeks after withdrawal; patient 15 had not achieved EASI-50 at the time of JAKi discontinuation. The 6 patients achieved EASI-50 16 weeks after switching to dupilumab, and 5 achieved EASI-75.

**Table 2** and **Figure 1** summarized the details regarding JAKi treatment status, disease control at 16 weeks after switching to dupilumab.

**Table 2:** Treatment status with JaKi and outcomes following switch to dupilumab.

Characteristics	Value [n (%)]
Type of JAKi	
Upadacitinib	9 (60.00)
Abrocitinib	6 (40.00)
Duration of JAKi’s treatment (week)	32.67 ± 18.79
Disease control upon discontinuation of JAKi	
EASI 75	10 (66.67)
PP-NRS ≤ 4	13 (86.67)
DLQI ≤ 5	11 (73.33)
ADCT<7	11 (73.33)
Reasons for switching to dupilumab	
Positive γ-interferon release test during JAKi therapy	4 (26.67)
Decreased efficacy after dose reduction and safety concerns	4 (26.67)
Bad efficacy	2 (13.33)
Safety concerns on the part of the doctors	2 (13.33)
Elevated transaminase	1 (6.67)
Elevated creatinine	1 (6.67)
Aggravated acne	1 (6.67)
Switch mode	
Switch immediately	9 (60.00)
Switch after discontinuing JAKi 2–20 weeks	6 (40.00)
Disease control after 16 weeks of dupilumab treatment	
EASI 75	11(73.33)
PP-NRS ≤ 4	14 (93.33)
DLQI ≤ 5	14 (93.33)
ADCT<7	14 (93.33)
Side effects	
JAKi	7 (46.67)
Dupilumab	0 (0.00)

JAKi, janus kinase inhibitors; EASI, eczema area and severity index; PP-NRS, peak pruritus numerical rating scale; DLQI, dermatology life quality index; ADCT, atopic dermatitis control tool.

## 4 Discussion

Here, we report 15 cases of moderate-to-severe AD patients switched from JAKi to dupilumab due to adverse events, inadequate efficacy, or safety concerns. Except for one patient who resumed upadacitinib, the remaining 14 achieved favorable outcomes. However, among the 9 patients who switched immediately, 4 experienced disease recurrence within the first 4 weeks after the switch. These findings suggest that dupilumab may be considered a potential alternative or maintenance therapy for patients with moderate-to-severe AD requiring discontinuation of JAKi, although attention should be paid to disease fluctuations during the early switching period.

Biologics and JAKi each possess distinct advantages in terms of clinical efficacy and safety profiles. JAKi, being oral formulations, offer convenient administration and faster onset of action compared to dupilumab, enabling rapid and high-level improvement in pruritus and clearance of skin lesions (7, 8); however, they are associated with increased risks of infections (particularly herpes infections), laboratory abnormalities and exacerbation of acne (1, 9–13). Dupilumab demonstrates superiority over JAKi in terms of long-term remission rates, efficacy stability, drug survival, and reduced relapse risk (14–21). It may also mitigates the risk of linear growth impairment in pediatric AD patients (22). Furthermore, dupilumab exhibits excellent long-term safety, with laboratory monitoring currently not considered necessary (23). Overall, oral JAKi hold advantages in terms of short-term efficacy and convenience. However, dupilumab excels in long-term maintenance and safety. Therefore, switching between the two drug classes according to their respective characteristics and individual patient conditions may maximize patient benefits.

Current drug switching predominantly occurs from dupilumab to JAKi, typically due to inadequate efficacy and/or adverse reactions. The majority of these patients who switch can achieve therapeutic goals and/or experience improvement in dupilumab-induced adverse events. Multiple studies have demonstrated that upadacitinib/abrocitinib can serve as effective treatment options for moderate-to-severe AD patients, regardless of prior response to dupilumab (24–26). Conversely, the response to biologic therapy following JAKi discontinuation remains poorly understood. Dupilumab demonstrates high safety and reduces the likelihood of disease recurrence, which is crucial for long-term patient health management. Therefore, exploring the potential of dupilumab as an alternative or maintenance therapy after discontinuing JAKi is warranted. Ollech A et al. reported that among 9 pediatric AD patients who switched to dupilumab after failing baricitinib treatment, 4 achieved complete remission and 4 achieved partial remission (27). Additionally, other researchers described 3 patients who achieved disease control with dupilumab after discontinuing upadacitinib/tofacitinib due to treatment failure or intolerance (28). In our cohort of 15 patients, the primary factors prompting the transition from JAKi to dupilumab included adverse reactions, unsatisfactory efficacy, and safety concerns. This discontinuation pattern is similar to the drug survival study conducted by van der Rijst et al. (29) in children with AD. Their study demonstrated that the 1-year, 2-year, and 3-year overall drug survival rates of dupilumab were significantly superior to those of methotrexate and cyclosporine. Meanwhile, the primary reasons for treatment discontinuation with cyclosporine and methotrexate were also poor efficacy and adverse reactions. Due to the short half-life of JAKi, discontinuation may lead to rapid loss of efficacy (30). One study showed that the median time to relapse of skin lesions with JAK inhibitors within 72 weeks after treatment discontinuation was 60 days (21), which was consistent with our observations. Among our patients, 5 experienced relapse within 2–20 weeks after discontinuing JAKi, and 4 experienced relapse during the first 4 weeks after an immediate switch from JAKi to dupilumab. Therefore, if an immediate switch from JAKi to a biologic is necessary, the possibility of temporary disease flare due to differences in half-life and onset of action should be considered. To minimize relapse during early switching period, the following transitional strategies may be considered: (1) short-term dual therapy, i.e., continuing JAKi for 2–4 weeks after immediate switching to dupilumab, to compensate for the relatively slow onset of action of dupilumab;(2) adding antihistamines, immunomodulators such as *Tripterygium wilfordii* Hook F, and intensifying topical treatments (topical corticosteroids or calcineurin inhibitors) during the first 4–8 weeks after switching, which helps control relapsing symptoms;(3) temporary transitional use of rapidly acting systemic agents such as cyclosporine for 2–4 weeks, with due consideration of their safety profile. Due to health insurance restrictions in our country (only one novel systemic agent can be reimbursable for moderate-to-severe AD within the same period), none of our patients received concomitant treatment. However, through short-term treatment with antihistamines, *Tripterygium wilfordii* Hook F, and topical corticosteroids while continuing dupilumab, symptoms were gradually controlled. Recently, an innovative study reported that through RNA sequencing of lesion skin scrapings, AD patients can be classified at the molecular level into a JAKi responder profiles and a Th2 molecular profiles. Those with the JAKi responder profile showed superior response to JAKi treatment, while those with the Th2 molecular profile exhibited no significant difference in efficacy between JAKi and Th2-targeted biologics (31). Based on this immune pathway-driven stratification research, we hypothesize that patients with Th2 molecular profile who experience severe pruritus and/or demand rapid relief of skin lesions but have concerns about long-term safety of JAKi, exhibit poor tolerance to JAKi may represent suitable candidates for switching from JAKi to dupilumab.

This study has inherent limitations. First, it is a single-center study, which limits the generalizability of the findings. Second, due to its retrospective design, small sample size, and heterogeneity in several aspects, the study has limited statistical power and a relatively high risk of bias. Furthermore, the absence of a control group precludes accounting for the influence of natural disease course. However, real-world switching scenarios can be highly complex and encompass a variety of clinical situations, and our study may provide insights that more closely reflect real-world clinical practice.

In conclusion, real-world evidence on switching from JAKi to dupilumab remains limited, and our findings offer preliminary real-world evidence for this treatment transition. We suggest that dupilumab may serve as a potential alternative or maintenance therapy for some patients after discontinuation of JAKi. However, frequent disease fluctuations may occur during the initial phase of immediate switching, necessitating close follow-up and proactive management. Nevertheless, current relevant research remains relatively scarce, and prospective, multi-center, large-scale studies are needed to confirm these findings.

## Data Availability

The original contributions presented in the study are included in the article/supplementary material. Further inquiries can be directed to the corresponding authors.
